# Neuroligin 4X overexpression in human breast cancer is associated with poor relapse-free survival

**DOI:** 10.1371/journal.pone.0189662

**Published:** 2017-12-15

**Authors:** Henry J. Henderson, Balasubramanyam Karanam, Rajeev Samant, Komal Vig, Shree R. Singh, Clayton Yates, Deepa Bedi

**Affiliations:** 1 Department of Biomedical Sciences, College of Veterinary Medicine, Tuskegee University, Tuskegee, AL, United States of America; 2 Department of Biology and Center for Cancer Research, Tuskegee University, Tuskegee, AL,United States of America; 3 Department of Pathobiology, The University of Alabama at Birmingham, Birmingham, AL, United States of America; 4 Center for Nanobiotechnology Research, Alabama State University, Montgomery, AL, United States of America; University of South Alabama Mitchell Cancer Institute, UNITED STATES

## Abstract

The molecular mechanisms involved in breast cancer progression and metastasis still remain unclear to date. It is a heterogeneous disease featuring several different phenotypes with consistently different biological characteristics. Neuroligins are neural cell adhesion molecules that have been implicated in heterotopic cell adhesion. In humans, alterations in neuroligin genes are implicated in autism and other cognitive diseases. Until recently, neuroligins have been shown to be abundantly expressed in blood vessels and also play a role implicated in the growth of glioma cells. Here we report increased expression of neuroligin 4X (NLGN4X) in breast cancer. We found NLGN4X was abundantly expressed in breast cancer tissues. NLGN4X expression data for all breast cancer cell lines in the Cancer Cell Line Encyclopedia (CCLE) was analyzed. Correlation between NLGN4X levels and clinicopathologic parameters were analyzed within Oncomine datasets. Evaluation of these bioinfomatic datasets results revealed that NLGN4X expression was higher in triple negative breast cancer cells, particularly the basal subtype and tissues versus non-triple-negative sets. Its level was also observed to be higher in metastatic tissues. RT-PCR, flow cytometry and immunofluorescence study of MDA-MB-231 and MCF-7 breast cancer cells validated that NLGN4X was increased in MDA-MB-231. Knockdown of NLGN4X expression by siRNA decreased cell proliferation and migration significantly in MDA-MB-231 breast cancer cells. NLGN4X knockdown in MDA-MB-231 cells resulted in induction of apoptosis as determined by annexin staining, elevated caspase 3/7 and cleaved PARP by flow cytometry. High NLGN4X expression highly correlated with decrease in relapse free-survival in TNBC. NLGN4X might represent novel biomarkers and therapeutic targets for breast cancer. Inhibition of NLGN4X may be a new target for the prevention and treatment of breast cancer.

## Introduction

Breast cancer is the most common cancer in women and is the second leading cause of cancer-related deaths. A median overall survival period of patients with this cancer remains 2 to 3 years [1]. Clinical management and treatment outcome in patients with breast cancer may vary due to its high heterogeneity at the histopathologic and molecular levels [2] as evident by clinicopathological characteristics and molecular markers. Breast cancer is a heterogeneous disease that has been classified into five major biologically distinct intrinsic subtypes: luminal A, luminal B, human epidermal growth factor receptor-2 (HER2) overexpressing, basal-like, and normal-like [3]. Despite advances in early detection and understanding of the molecular basis of breast cancer biology, about 40% of the patients with early-stage breast cancer have recurrent and metastatic disease [4]. Improving our understanding of the molecular mechanisms of the metastatic process might also improve clinical management of the disease. Tumor metastasis consists of a complex series of events including cell migration, invasion, adhesion and blood vessel formation. Initiation of metastasis requires invasion, which is enabled by epithelial to mesenchymal transition of cancer cells. The process of tissue invasion and metastasis involves a series of attachment and detachment events based on cell or substrate attachment [5]. One crucial step during tumor invasion is loss of cancer cells adhesiveness to the extracellular matrix component of basement membrane and mesenchymal tissue. It is believed that these invasive cells have undergone an epithelial to mesenchymal transition (EMT), which is associated with increased expression of cell-adhesion molecules such as laminin, α6β4 integrins, and CD44 [6]. Cell junctions like adherens, septates and tight junctions play an important role in the control of cell proliferation, intercellular barrier formation, cellular differentiation, survival, apoptosis and angiogenesis [7]. Cell-adhesion molecules such as ICAM, CD146, and the glycoprotein NMB play an important role in mediating metastasis [8–10]. Neuroligins constitute a family of neuronal transmembrane synaptic proteins whose structural and biochemical characteristics are indicative of a role in heterotypic cell adhesion [11, 12]. The neuroligin (NLGN) gene family consists of five members (NLGN1 at 3q26, NLGN2 at 17p13, NLGN3 at Xq13, NLGN4 at Xp22, and NLGN4Y at Yq11) [13]. Their large extracellular N-terminal domain is homologous to serine esterases. They are of great importance in mediating synapse formation in the central nervous system, and they interact with neurexins from the opposite side (in trans) of the synaptic cleft in a calcium-dependent manner [14]. Both proteins display a strong and selective synapse formation which promotes activity between neurons *in vitro*. When expressed in non-neuronal cells, they mediate synaptogenesis between the cells and the adjacent neurons [15, 16]. Besides being neuronal proteins, neuroligins have been implicated in vascular remodeling during angiogenesis [17]. Recent studies have shown a mitogenic effect of NLGN3 in glioma proliferation and progression [18]. Recent studies highlight the role of NLGN1 in stabilizing laminin interaction with integrins suggests the importance of this molecule in mediating cell adhesion. Other studies have shown that NLGN1 is overexpressed in a metastatic model of prostate cancer and is responsible for cell adhesion [19]. Our previous studies using a selection from a combinatorial random peptide library against breast and pancreatic cancer cell lines identified several peptides [20] mimicking neuroligin (NLGN-1, 3 and NLGN4X). In this study, we sought to determine the expression of NLGN4X, its relevance and functional significance in breast cancer.

## Materials and methods

### Cell lines and culture conditions

All cell lines were obtained from American Type Culture Collection(ATCC). MCF-7 and MDA-MB-231 breast cancer cells were maintained in Dulbecco's modified Eagle's medium supplemented with 10% fetal bovine serum (Sigma) at 37°C in an incubator containing 5% CO2 and humidified air.

### Public Dataset: Cancer Cell Line Encyclopedia (CCLE) data analysis

The data was accessed at the following URL: http://www.broadinstitute.org/ccle/home. Using the GENE-E analysis tool on the website, the expression dataset of all breast cancer cell lines was downloaded.

### Oncomine analysis

Oncomine (Compendia Bioscience, Ann Arbor, MI) was used for analysis and visualization of the TGCA annotated breast cancer datasets (http://www.oncomine.org). NLGN4X RNA expression levels were displayed using log2 median centered ratio boxplots for non-metastatic vs. metastatic profile.

### cBioportal

The Breast Invasive Carcinoma (TCGA-provisional) data set (959 samples) was queried at cBioportal (www.cbioportal.org) for NLGN4X DNA copy number alteration.

### Kaplan–Meier curve

Kaplan–Meier curves were generated with the KMplot software, using a database of public microarray datasets [21]. Altogether, results were collected from 3,557 patients. Out of these patients, 2036 were ERα-positive and 806 ER-negative, by immunohistochemistry. Kaplan–Meier plots were generated after averaging the probes.

### Tissue specimen and immunohistochemistry

The breast tissue microarrays were obtained from Novus Biological (Littleton, CO). These include 40 breast cancer infiltrating ductal carcinoma, 10 metastatic lymph node and 9 adjacent normal breast tissues. The use of tissues was approved by the Institutional Review Board of Tuskegee University. Clinicohistopathologic characteristics of the subjects in the tissue microarray study included age, grade, hormone status and clinical stage, according to information provided by the suppliers. The expression levels were classified as negative (≤0.3), weak positive (0.3 to 1.5), or strong positive (≥1.5). Tissues were de-paraffinized in xylene and rehydrated in graded alcohols. For antigen retrieval of NLGN4X the slides were pressure-cooked for 10 minutes. Endogenous peroxidase activity was quenched with 3% hydrogen peroxide for 5 minutes. Slides were blocked by 3% goat serum at room temperature for 1 hour in humidity chambers with NLGN4X antibody (1:100) (Gentex, CA).The HRP conjugated goat anti-mouse/anti-rabbit secondary antibody (Jackson Immunoresearch Laboratories Inc, West Grove, PA) was applied for 40 minutes. The antigen-antibody reaction was visualized after diaminobenzidine (Sigma-Aldrich, MO) was applied for 7 minutes. The slides were counterstained with hematoxylin (Sigma-Aldrich, MO) for 1 minute. Positive controls were included in each staining run; negative controls were obtained by omitting the primary antibody. Slides were then dehydrated in alcohols and cleared in three xylene baths before being mounted with permount media.

### NLGN4X knockdown

siRNAs for human NLGN4X and scrambled siRNA were purchased from Integrated DNA Technologies (IDT, Coralville, Iowa, USA). Human MDAMB231 cells were transfected with Lipofectamine RNAiMAX (Invitrogen) according to the manufacturer’s protocol. To study NLGN4X gene knockdown, 10^5^ MDAMB231 cells in 6-well culture plates were transfected with NLGN4X-specific siRNA (UGAGAGAUCCUUACUGCAUGACATG,AAGUAUCCAAAUUGGCGGUAAACCAGA,CCAAUCGAUGUUUAGUGUGAUAGGA) (40 nM) or mixed with Lipofectamine RNAiMAX (Invitrogen, Carlsbad, CA). The plates were rocked gently at room temperature and incubated at 37°C for 24–72 h. The medium was changed every 24h.

### Western blot analysis

Cells were lysed in ice-cold complete 1x RIPA buffer (PMSF solution, sodium orthovanadate solution, protease inhibitor cocktail solution, and 1x lysis buffer) (Santa Cruz Biotechnology, Santa Cruz, Ca). The proteins were then quantified using the BCA Protein Assay Kit (Pierce Biotechnology, Rockford, IL). 40 μg of protein from each sample was separated by a 4–12% SDS-PAGE gel and then transferred to a 0.2 μm polyvinylidene difluoride(PVDF) membrane. Membranes were first blocked with 5% nonfat dry milk in TBS-T and then incubated with the NLGN 4x primary antibody (1:1000) overnight at 4°C. After washing, membranes were incubated with horseradish peroxidase(HRP)-conjugated secondary antibody. Membranes were washed and blots were visualized using enhanced chemiluminescence. To verify that equal amounts of protein was loaded from each sample, the membrane was stripped with mild stripping buffer and reprobed with β-actin(Cell Signaling,Danvers, MA). Relative levels of gene expression were quantified using the imager (Image J Image Processing Software).

### Analysis of NLGN4X gene expression by RT-PCR

Total RNA was extracted using the RNeasy Micro kit (QIAGEN GmbH, Hilden, Germany) according to the manufacturer’s protocol. Reverse transcription of total RNA and cDNA amplification by PCR was carried out using 25 ng of total RNA using one step Access RT-PCR kit according to the manufacturer’s protocol. The primers for NLGN4X 5’CCCAATGAAATCTTGGTCCGTG3’ and 5’CTGAGGGTC ATCTGGAATCACATCTC 3’ and *GAPDH* 5’ CATGTTCGTCATGGGTGTGAA 3’ and 5’ AGTGATGGCATGGACTTGGT 3’ genes were used at final concentration 0.1 °M. One cycle of reverse transcription of isolated RNA at 48°C (45 min) and 94°C (2 min) was followed by 40 cycles of PCR at 62°C (30 sec), 68°C (1 min) and 68°C (7 min). Relative levels of gene expression were quantified using the imager (Image J Image Processing Software).

### Flow cytometry analysis

MDA-MB-231 and MCF-7 cells were grown in 25cm^2^ flask and were trypsinized upon 80% confluency. Next, cells were brought to a concentration of 10^5^ cells per 100 μl of blocking buffer (PBS containing 1% BSA and 1% triton-X100), followed by incubation with anti–NLGN4X antibody (1:100), anti-cleaved PARP, anti-caspase 3 and anti-caspase 7 (Cell Signaling technology, Ma) for 1 h at room temperature (RT). Cell were washed 3 times through suspension–spin cycles and incubated with Alexa-flour anti-rabbit secondary antibody for 1 hr at RT. Cells were washed twice in washing buffer (PBS–1% BSA, 1% Triton-X-100 and 0.1% Tween-20) and then incubated with a secondary goat anti-mouse antibody conjugated with Alexa fluor 488 (Life Technologies,CA) for 45 min at RT. Following 3 washes, cells were suspended in 100uL of PBS and evaluated by flow cytometry (Bectpn Dickinson FACSCalibur). For cells treated with siRNA, experiments were performed 72hrs post-transfection.

### NLGN4X immunofluorescent staining

Cells (1 × 10^5^ cells) were seeded in a Lab-Tek II Chamber Slide System separately with 1 mL medium overnight at 37°C. After medium was removed, cells were rinsed with PBS three times and fixed with 4% formaldehyde in PBS at RT for 10 min, followed by washing with PBS. Samples were blocked with 1% BSA in PBS for 30 min in an ice bath. After BSA blocking, samples were stained with mouse anti-human NLGN4X antibody (primary antibody) for 1 h and rinsed with PBS. Samples were incubated with Alexa fluor 488 conjugated goat anti-rabbit secondary antibody (Life Technologies,CA) for another 1 h, followed by washing with PBS. DAPI was used to stain the cell nucleus. Samples were examined under a Leica TCS SP5 confocal fluorescent microscope (Leica Microsystems). Digital images were captured with AxioVision digital image-processing software.

### Wound-healing cell migration assay

Cell migration assays included cells with NLGN4X and cells with NLGN4X knocked down by siRNA. Briefly, cells (2 × 10^5^) were seeded in 6-well plates and allowed to form an 80% confluent monolayer. Cells were cultured in serum-free medium overnight before wounding. The wound was made by the tip of a 200-μL micropipette passed across the monolayer. After 48 hours, the distance that cells moved was determined and quantified in Metamorph imaging software. Shown are the mean values ± SEMs of 3 measurements for each time point and condition. All measurements were normalized to 0-hour controls.

### Cell viability assay

Cell proliferation in MDA-MB-231 was detected using an MTS assay. Transfected and control cells were plated in 96-well plates at 5×10^3^ cells/well and cultured in DMEM for 24, 48 and 72 h. The culture medium was then replaced with 100 μL of fresh DMEM followed by the addition of 20 μL of MTS solution (CellTiter 96 Aqueous Non-Radioactive Cell Proliferation Assay (Promega, Madison, WI, USA) to each well. Plates were incubated at 37°C for 4 h. At these time points, the culture medium was replaced followed by the addition of 20 μL of MTS (5 mg/ml) for another 4 h at 37°C. The optical density (OD) at 490 nm was determined with microplate reader (BioTek).

### Annexin V-FITC apoptosis detection by flow cytometry

MDA-MB-231 cells were seeded at 150,000 cells/35-mm cell culture dish and transfected with NLGN4X siRNA (40nM) to deplete NLGN4X. Cells were thereafter grown for 72h. At 72 h post transfection, apoptosis of MDA-MB-231 cells was detected by flow cytometry. Subsequently, Apoptosis was measured using the Annexin V-FITC Apoptosis Detection Kit (Sigma-Aldrich™, Saint Louis, MO,USA). Following a 72-hour siRNA Transfection period, cells were trypsinized and centrifuged to collect the pelleted sample. Cells were then suspended in 500ul of 1x Binding Buffer at a concentration of ~ 1 x 10^6^ cells/ml. The cell suspension was then transferred to a plastic 12 x 75 mm test tube. 5ul of Annexin V FITC Conjugate and 10ul of Propidium Iodide Solution were added to each cell suspension. The tubes incubated at room temperature for exactly 10 minutes and were protected from light. Fluorescence of the cells was determined immediately with a flow cytometer.

### Statistics

The significance of difference between two variables was assessed by the Student's t test. The difference was considered significant if the p value was <0.05. Data from all experiments are expressed as mean ± standard error mean (SEM).All statistical calculations were performed using GraphPad Prism and Microsoft Excel.

## Results

### Cancer-selective phages identified a unique set of sequences related to Neuroligin’s

**Fig 1:**
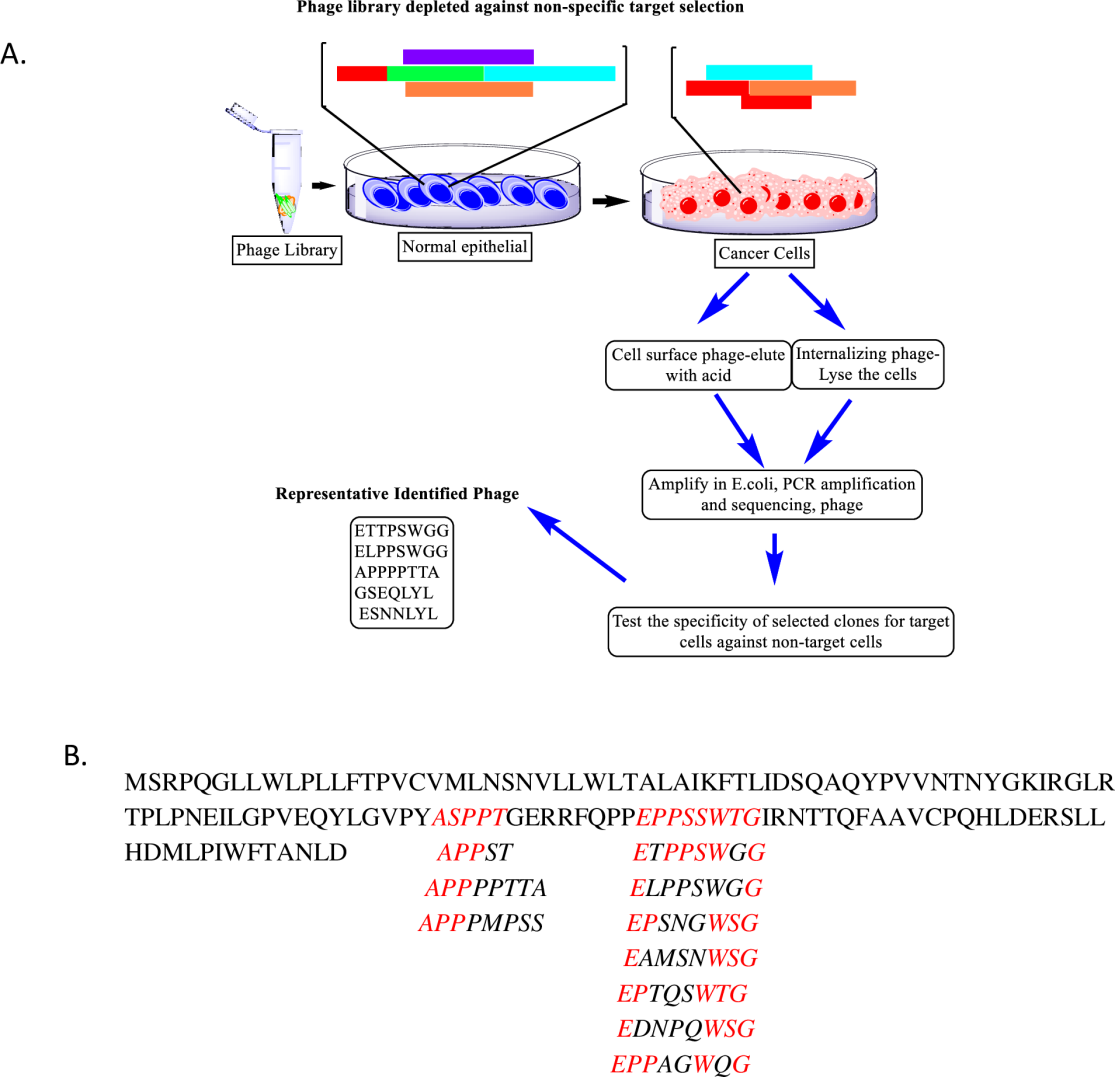
Phage selection and sequence similarity to NLGN4X. A) Flow chart depicting the selection of phages from the landscape phage library against cancer cells. B) Amino acid sequences of phages selected on human breast cancer cells (red) matched to the sequence of human Neuroligin 4X.

Previously, through screening of the combinatorial landscape phage library, we identified several phages binding to pancreatic and breast cancer cells, PANC-1 and MCF-7, respectively, as shown in Fig 1 [20]. As described previously, a family of phages-derived peptides with motif E—PSW—and APP—exhibited high affinity and specificity to breast cancer cells. A sequence similarity search for proteins mimicked by these phage peptides performed with BLAST identified the E—PSW—and APP—motif present in peptides to be similar to a conserved sequence within the neuronal cell adhesion molecule, neuroligins 1, 3 and 4X (Fig 1B). Neuroligin 4X is located in the X chromosome, specifically at Xp22. Xp22 locus is particularly rich in escape genes [22] and dysregulation of certain genes in this Xp22 region is an important feature of basal-like breast cancer [23]. Therefore, we decided to focus on the role of NLGN4X in breast cancer.

### NLGN4X is widely expressed in breast cancer cells

**Fig 2:**
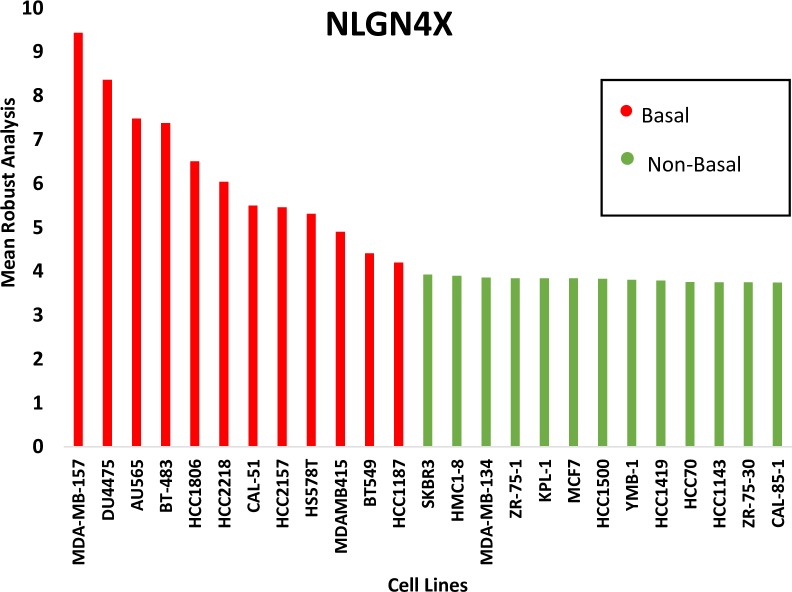
NLGN4X is highly expressed in breast cancer cell lines, particularly TNBC. The figure depicts Mean Robust Analysis of NLGN4X mRNA expression across multiple cell lines in the CCLE database.

**Fig 3:**
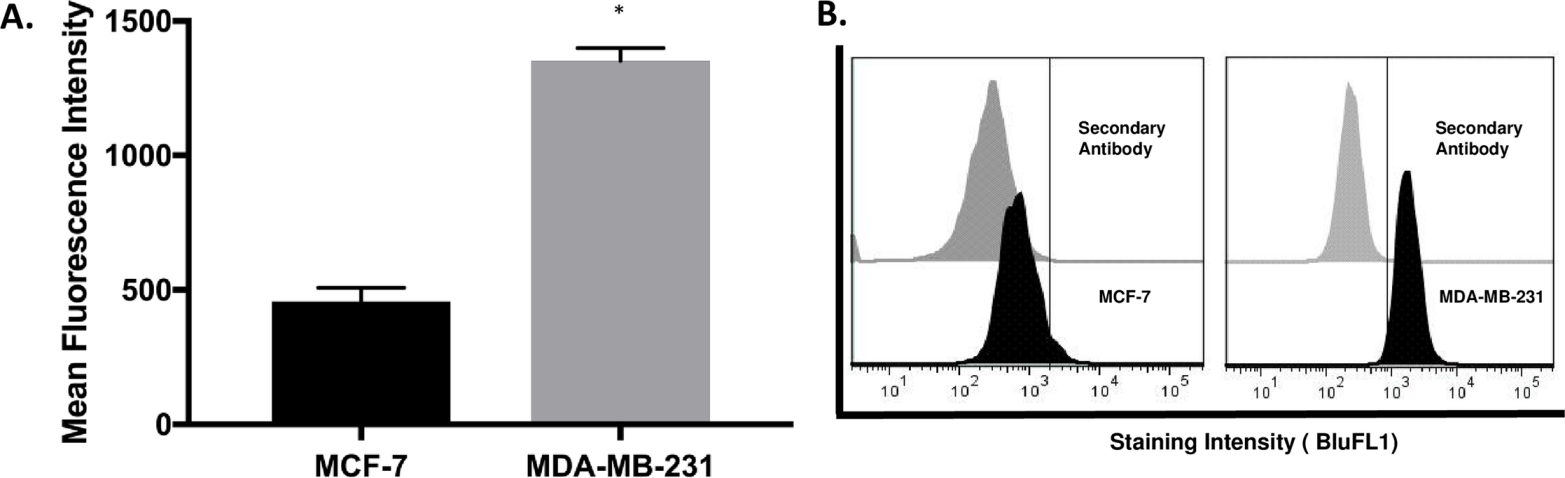
NLGN4x expression in selected breast cancer cell lines. A) NLGN4X protein expression in two different cell lines MCF-7 and MDA-MB-231, as determined by measuring mean fluorescence intensity by flow cytometry. B) The fluorescence curves of secondary antibody intracellular staining and NLGN4X mAB intracellular staining for the cell lines stated above.

**Fig 4:**
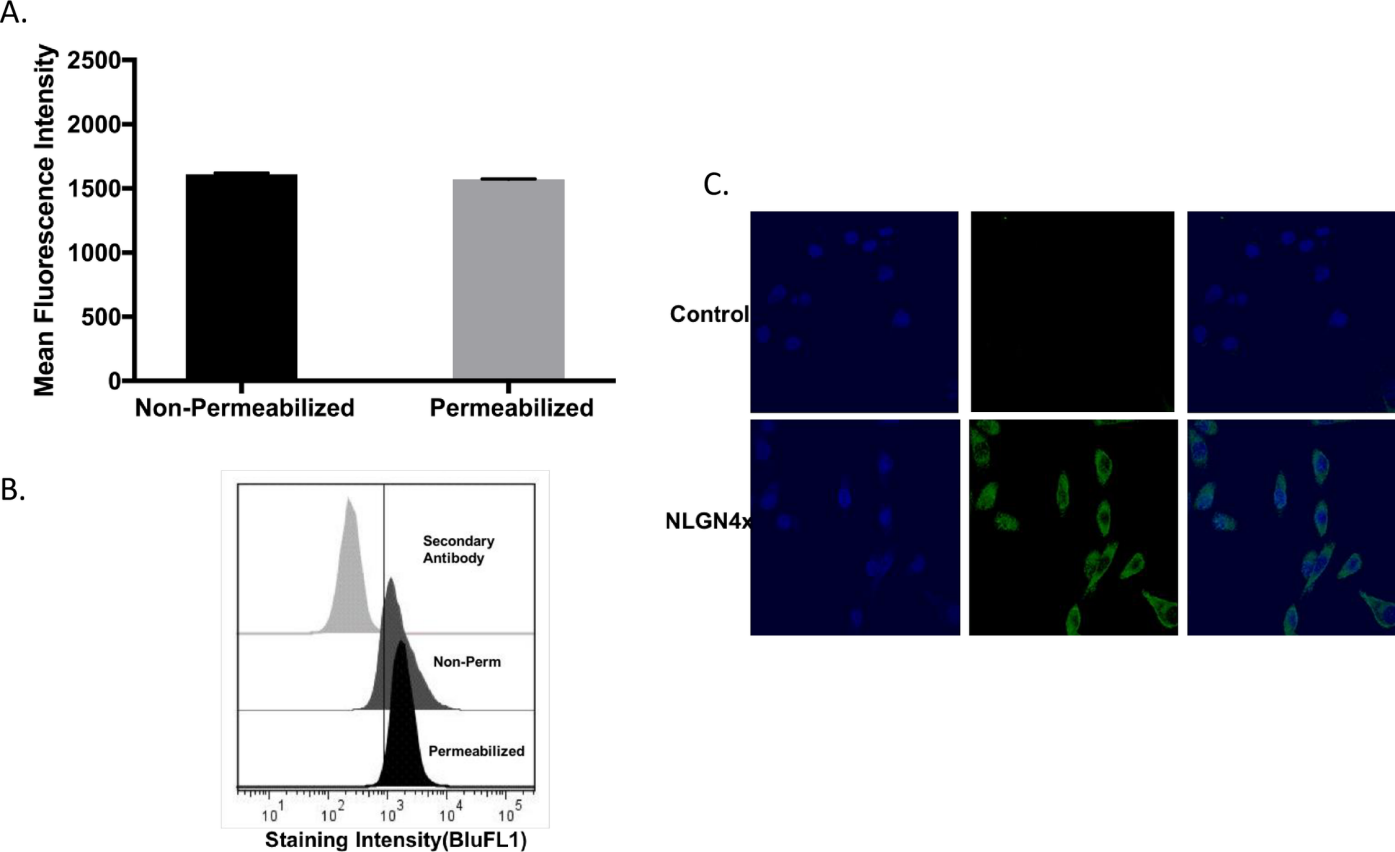
Cellular localization of NLGN4X. A) Cell surface staining of NLGN4X in non-permeabilized and permeabilized MDA-MB-231 by measuring mean fluorescence intensity as determined by flow cytometry. B) The fluorescence curves of secondary antibody intracellular staining and NLGN4X mAB intracellular staining for the non-permeabilized and permeabilized MDA-MB-231; C) Representative images of NLGN4X staining in MDA-MB-231 as visualized by Confocal IF Control denotes secondary antibody staining without NLGN4X primary antibody.

The gene expression data for all the breast cancer cell lines within the Comprehensive Cell Line Encyclopedia (CCLE) database was studied to see whether the pattern of NLGN4X expression could be confirmed in a larger dataset. The mean Robust Mean Analysis (RMA) value of NLGN4X (Fig 2) expression in the group featuring TNBC (MDAMB157, HCC1806, Cal-51, HDQ-P1) was significantly greater than the hormone-positive-type (SKBR-3, MDA-MB-134, MCF-7) (6.1 vsus 3.5, p = .002). The phenotype and receptor subtype of these cell lines are listed in the Table 1. We used the cBioPortal database to evaluate the transcriptional expression from breast cancer tissue and observed that NLGN4X was altered in 25 out of 1080 patients. To determine if mRNA data from CCLE correlated with protein expression, 2 breast cancer cell lines, MCF-7 and MDA-MB-231 were analyzed for protein expression by flow cytometry(Fig 3). MDA-MB-231 showed the highest expression of NLGN4X as compared to MCF-7(Fig 3A). Flow cytometry analysis of non-permeabilized MDA-MB-231 cells revealed that NLGN4X was present on the cell surface (Fig 4A and 4B). Confocal microscopy of the MDA-MB-231 cells revealed the presence of NLGN4X on the plasma membrane as well as in the cytoplasm (Fig 4C).

**Table 1:** Assignment of NLGN-4X-expressing cell lines with phenotype and receptor status.

Cell lines	Phenotype/subtype	Receptor
MDA-MB-157	Epithelial/Basal	TNBC
DU4475	Basal	TNBC
AU565	Epithelial/Luminal	hrprher+
HCC1806	Basal	TNBC
HCC2218	Epithelial/Luminal	er+her+
CAL-51	Mesenchymal/Basal	TNBC
Hs 578T	Mesenchymal/Basal	TNBC
MDA-MB-415	Epithelial/Luminal	er+
BT549	Post-EMT	TNBC
HCC1187	Epithelial/Basal	TNBC
MDA-MB-134	Epithelial/Luminal	er+
ZR-75-1	Epithelial/Luminal	er+
KPL-1	Epithelial/	er+pr-her2-
MCF7	Epithelial/Luminal	hrprher+
HCC1500	Epithelial/Basal	hrpr+ her-
HCC1419	Epithelial/Luminal	er+her+
ZR-75-30	Luminal	er+her+

### NLGN4X is expressed in human breast carcinoma

**Fig 5:**
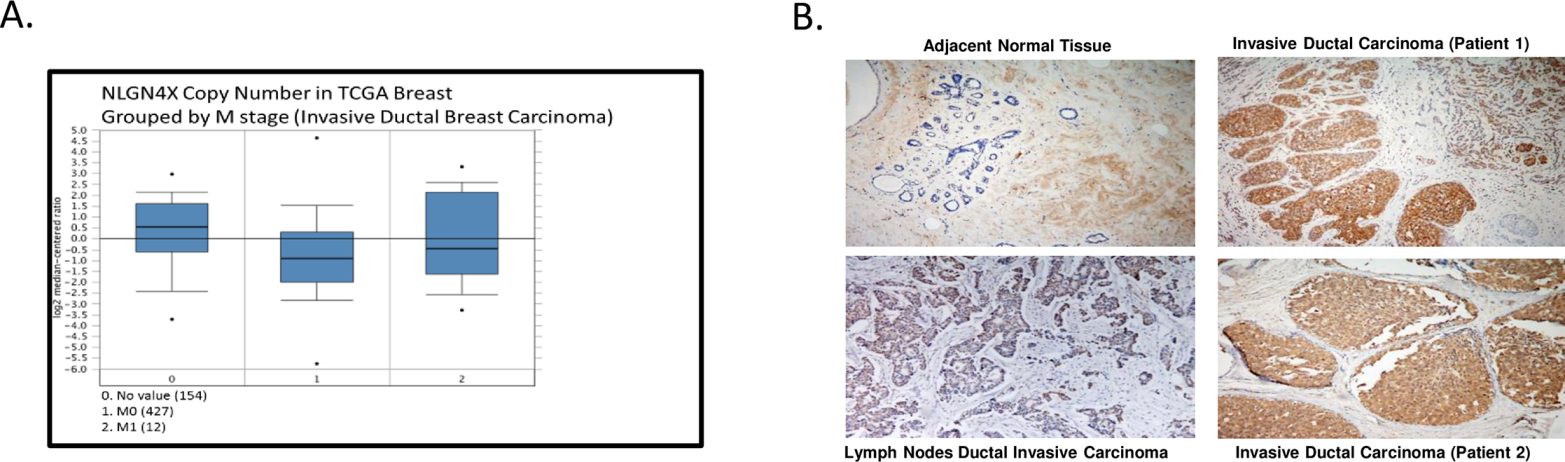
NLGN4X is expressed in breast cancer tissues and metastasis. A) Oncomine box plot RNA expression data for NLGN4X in metastatic versus non-metastatic are shown within the TCGA data set. B) Representative image of breast cancer microarrays showing NLGN4X expression in normal, invasive ductal carcinoma and lymph node of invasive ductal carcinoma.

Using the bioinformatics oncomine database, we correlated NLGN4X expression and metastatic potential. TGCA datasets showed increased median expression of NLGN4X in metastatic tumor sites compared to primary sites, as shown in Fig 5A. The metastatic sites where its expression was higher were bone, liver and brain (data not shown). To evaluate the expression and localization of NLGN4X in breast cancer tissues, immunohistochemistry was used to evaluate samples from 60 patients, consisting of adjacent normal tissue (9 patients), breast cancer tissues tumors (40) and respective lymph node metastasis (10). There was low expression of the NLGN4X protein in noncancerous samples (Fig 5B); and high expression in carcinoma (Fig 5B); expression was predominantly in the membrane or cytoplasm. NLGN4X staining was also seen in respective lymph node metastasis. The tissues were scored for NLGN4X staining with intensities ranging from 0 to 3 between normal and cancerous tissue by pathologists.

### NLGN4X expression is correlated with survival

**Fig 6:**
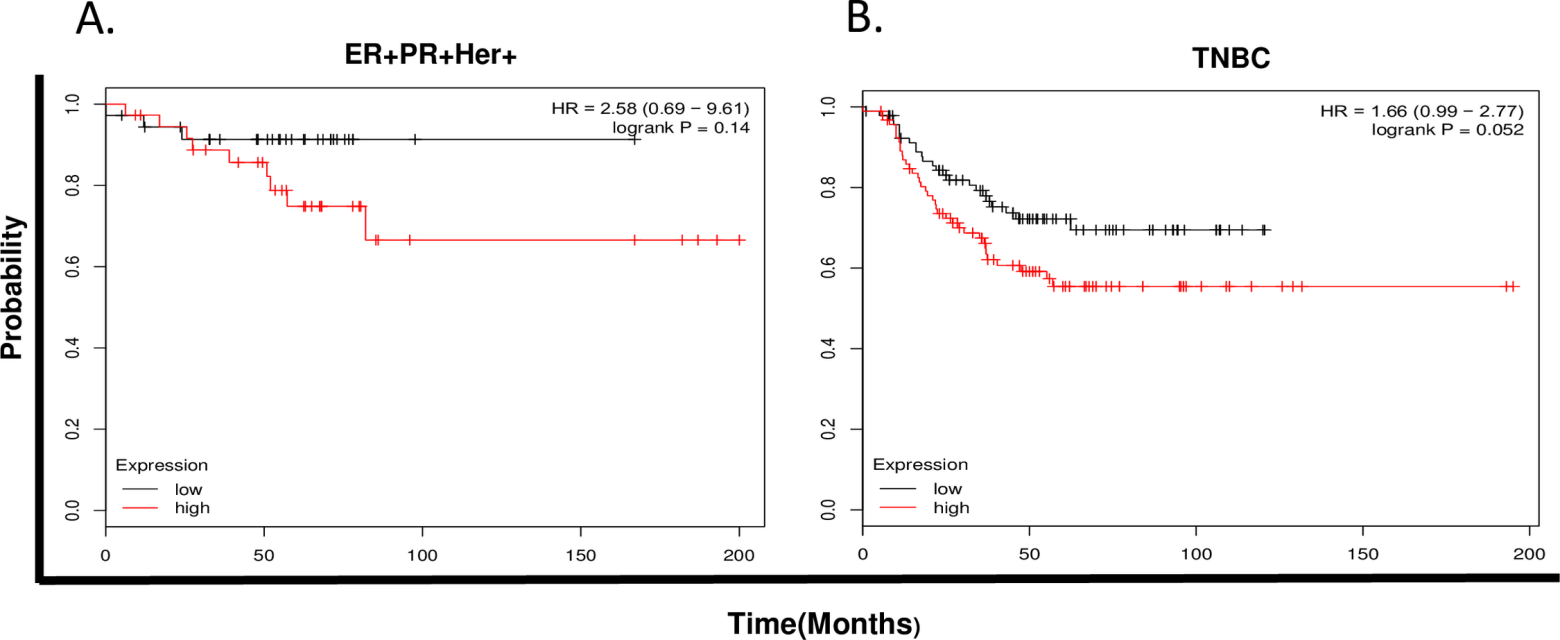
NLGN4X mRNA correlates with relapse-free survival. Kaplan-Meier survival plots demonstrate the prognostic relevance of NLGN-4X expression on patient survival using various data cohorts. High NLGN-4X expression correlated with decrease in survival in A)ER, PR and Her2+ tumors and B)TNBC.

Using a Kaplan–Meier analysis approach against a database of publicly available breast cancer samples, we demonstrated that higher NLGN4X (Fig 6) in ER+, PR+, Her+ and hormone receptor-negative basal-type breast cancer correlated significantly with a decrease in relapse-free survival and predicts advanced tumor malignancy and a poorer prognosis.

### NLGN4X knockdown by siRNA inhibits breast cancer migration and proliferation

**Fig 7:**
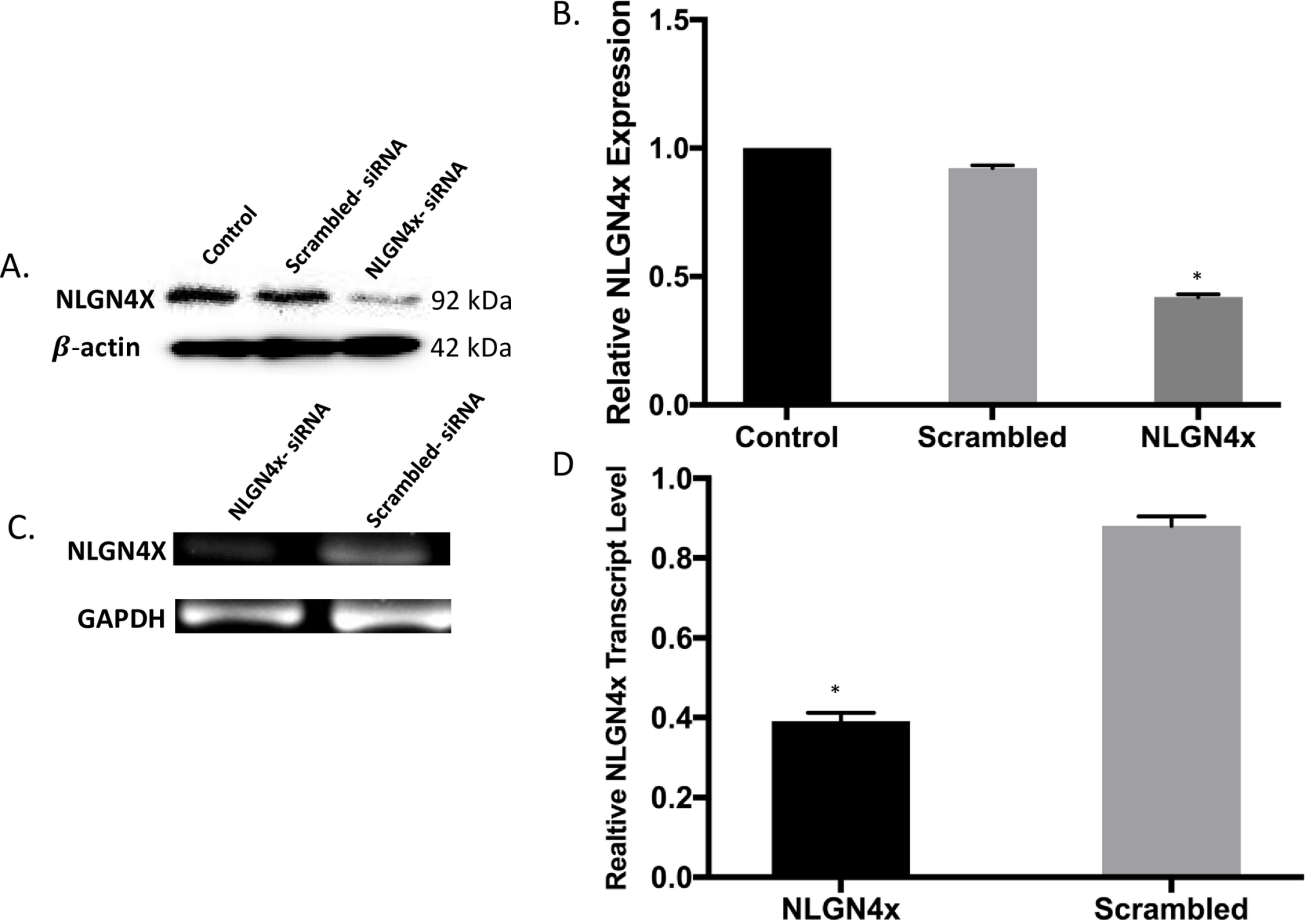
Analysis of NLGN4X gene knockdown. A) The protein level of NLGN4X in MDA-MB-231 as determined by western blot. B)Relative protein expression was normalized against β-actin. C)Transcription level of the target gene in MDA-MB-231 cells treated with NLGN4X-siRNA or Scrambled siRNA by RT-PCR. D) The relative quantification was normalized against GAPDH. Normalizations for both analyses were conducted with IMAGE J image analysis software. All data represent the mean± S.D. *p <0.05, student-t-test. All data represent the mean± S.D. * p <0.05, student-t-test.

**Fig 8:**
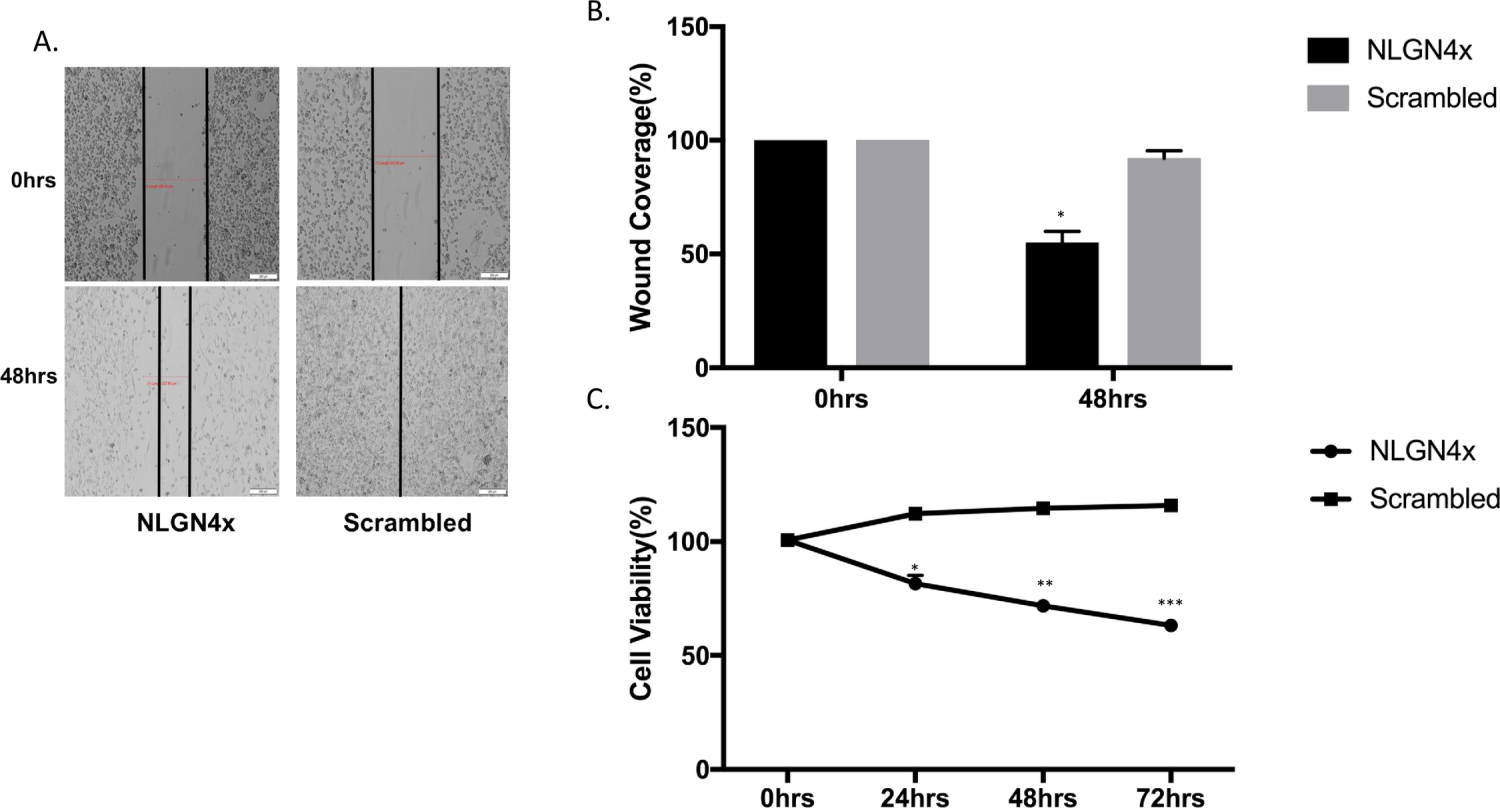
Effect of NLGN4X on cell migration and proliferation. A). Quantification of area migrated in NLGN4X knockdown MDA-MB-231 cells compared with scrambled control. Migration is expressed as the percentage of wound coverage area (mean ± SD). B) Representative images are shown (Magnification, x200. Scale bars, 40 μm). Data are representative of three independent experiments (***p<0.001 t test). C) Effect of NLGN4X knockdown on proliferation of MDA-MB-231 cells. Graph represented OD at 490 nm ± SD, p < 0.05. Data are representative of at least three independent experiments.

NLGN4X was effectively knocked down as determined by western blot (Fig 7A) and RT-PCR (Fig 7C). The effect of NLGN4X on cell migration post transfection was evaluated. Fig 8A and 8B indicates that MDA-MB-231 cell migration is significantly decreased 48 h after siRNA transfection. The relative migration rate of cells transfected with NLGN4X siRNA group was significantly lower compared to the scrambled siRNA groups. The time-dependent effect of NLGN4X knockdown on cell proliferation was evaluated by MTS assay. The results obtained indicated that MDA-MB-231 cell proliferation was significantly decreased by 20%, 30% and 50% in the NLGN4X siRNA groups 24h, 48 h and 72 h, respectively post transfection as compared to cells transfected with scrambled siRNA (Fig 8C).

### NLGN4X knockdown causes apoptosis in breast cancer

**Fig 9:**
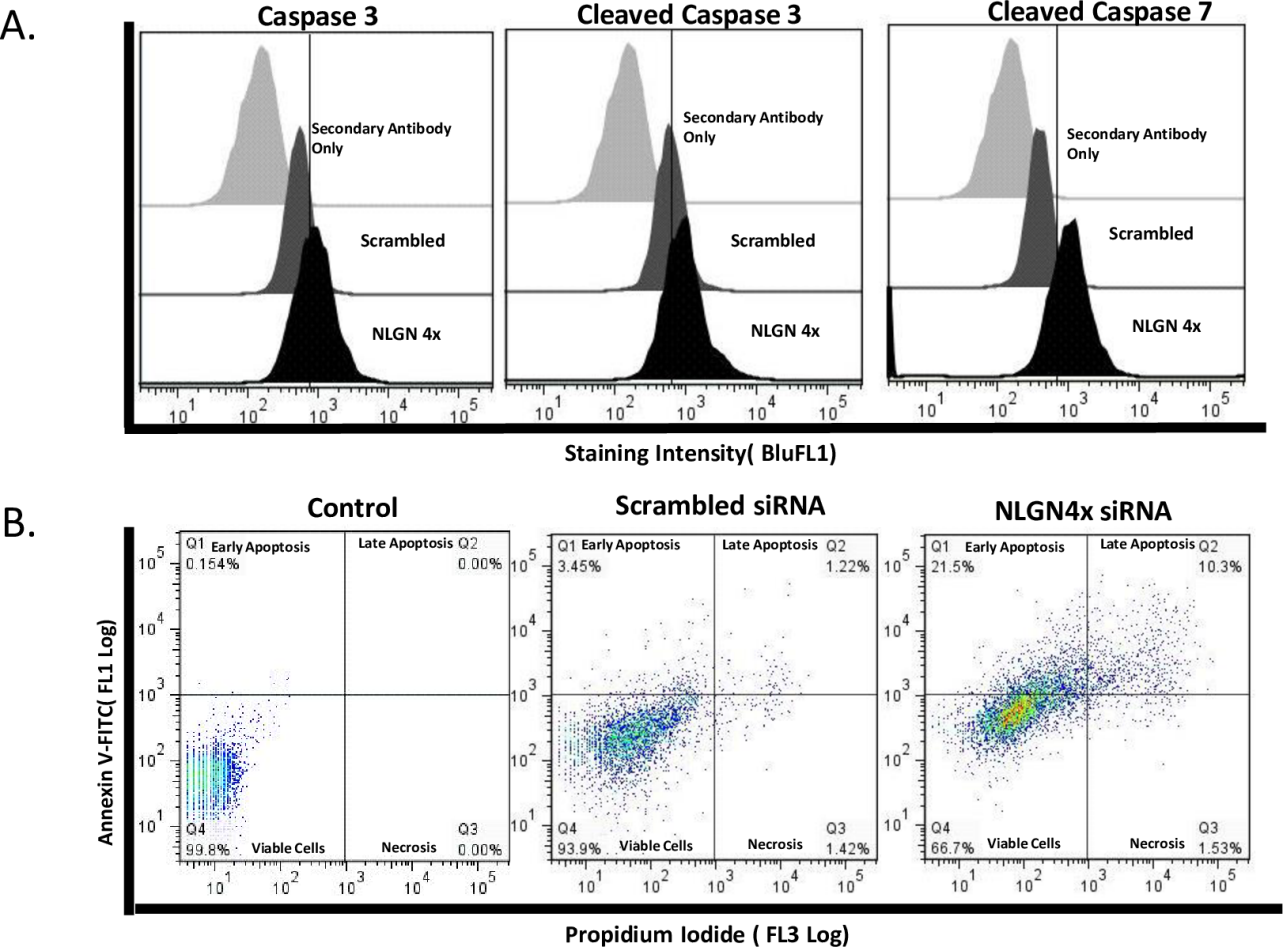
Effect of NLGN4X knockdown on apoptosis and apoptotic markers. A) The fluorescence curves of secondary antibody intracellular staining, then apoptosis markers caspase 3, 7 and cleaved PAPRP mAB intracellular staining in siRNA-treated vs scrambled-siRNA treated cells. B.) Apoptotic activity was analyzed on a flow cytometer with quadrants designating Early Apoptosis, Late Apoptosis, Necrosis, or Viable Cells in relation to NLGN4X siRNA and scrambled sirNA treated cells.

As shown in Fig 9A, NLGN4X knockdown resulted in higher population of early apoptotic population (21.3% in MDA-MB-231 cells) compared to control and scrambled siRNA (<4%). Flow cytometry analysis of MDA-MB-231 cells transfected with NLGN4X or scrambled siRNA with caspase 3, caspase 7 and cleaved PAPRP revealed NLGN4X siRNA significantly increased caspase 3, 7 and cleaved PARP as compared to scrambled siRNA suggesting that inhibition of proliferation mediated by knockdown of NLGN4x was in part caused by induction of apoptosis. To examine whether cells undergo apoptosis, NLGN4X siRNA transfected or scrambled siRNA transfected MDA-MB-231 breast cancer cells were stained with annexin V and PI. Flow cytometry analysis of stained cells can distinguish cells into four groups, namely viable (annexin V- PI-), early apoptosis (annexin V+ PI-), late apoptosis (annexin V+ PI+) and necrotic (annexin V- PI+) cells (Fig 9B).

## Discussion

Among American women, breast cancer is the most common cancer and is the second leading cause of cancer death. It is a very heterogeneous disease, exhibiting various subtypes. Among the subtypes, basal-type represents the most aggressive type, with high metastatic potential [24]. Improving our understanding of the molecular mechanisms of the metastatic process may improve clinical management of the disease. A growing body of evidence indicates that alterations in the adhesion properties of neoplastic cells play an important role in the development and progression of cancer. Cell adhesion molecules provide a mechanistic role in the process of cancer metastasis and invasion [25, 26]. The metastatic event involves a series of consecutive attachment and detachment events that are based on cell-to-cell and cell-to-substrate interactions. Invasive tumor cells’ ability to reach the blood, lymph nodes, and distant organs depends highly on the adhesive interaction with the basement membrane and extracellular matrix of the target host organ. Several cell adhesion molecules such as ICAM 1, laminin and fibronectin have been associated with the invasive process, growth, and metastasis of breast tumors. [27]. This is the first study to link the expression of neuronal cell adhesion molecules, neuroligins, to breast cancer. Here we show that the expression of NLGN4X is significantly higher in several subset of breast cancer, according to the CCLE database. This was further confirmed with flow cytometry analysis on the two selected cell types that are representative of different breast cancer cell subtypes and that express a range of NLGN4X; MCF-7 (ER+/PR+/HER2−/low NLGN4X) and MDAMB231 (ER−/PR−/HER-/very high NLGN4X). Immunofluorescence confocal microscopy of MDA-MB-231 cells with anti-NLGN4X antibody again confirmed the presence of NLGN4X. The bioinformatics Oncomine TGCA data revealed that NLGN4X expression was increased in metastatic tissue. It is possible that NLGN4X can modulate the metastatic behavior of breast cancer cells, and possibly play a role in the ability of cancer cells to metastasize to distant sites. These observations are supported by the fact that NLGN4X expression predicts relapse-free breast cancer survival outcomes in patients with the disease. Our IHC data on breast cancer TMA shows NLGN4X is minimally present in adjacent normal breast tissue while that high NLGN4X expression was observed in breast carcinoma and lymph node metastasis. 30 out of 40 and 6 out of 10 breast cancer tissues and lymph node metastasis, respectively were positive for NLGN4X. We did not find any difference in staining of NLGN4X in ER+, PR+ vs ER-, PR- breast cancer tissues.

NLGN4X protein and mRNA was effectively down-regulated in MDA-MB-231 cells after siRNA transfection. The NLGN4X siRNA-transduced cells showed inhibition in cell proliferation and migration in MDA-MB-231, indicating the migration and proliferation of TNBC cells were regulated by NLGN4X expression. The decrease in proliferation was mediated by induction of apoptosis by NLGN4X knockdown as evident by elevated levels of cleaved PARP and caspase 3/7 activity. The depletion of NLGN4X also resulted in an increase of cells in both the early stages and late stages of apoptosis. These suggest the role of NLGN4X as being essential for cancer cells survival.

Based on these data, it is a good possibility that and NLGN4X can be used as a biomarker for breast cancer and for prediction of survival outcomes of patients with hormone positive, Her+ subtype and ER-, basal subtype. To the best of our knowledge, this is the first study to evaluate the prognostic significance of neuroligins expression in breast cancer. Further detailed studies are required to elucidate the mechanistic role of these adhesion proteins in breast cancer progression and metastasis.
